# Infantile Acute Myeloid Leukemia Presenting As Intussusception: A Case Report and Review of the Literature

**DOI:** 10.7759/cureus.96237

**Published:** 2025-11-06

**Authors:** Yuanyuan Gu, Christopher Blackmore, Kathryn McFadden, Ketan Kulkarni

**Affiliations:** 1 Department of Pathology and Laboratory Medicine, Queen Elizabeth II Health Sciences Centre, Dalhousie University, London, CAN; 2 Department of Surgery, IWK Health Centre, Halifax, CAN; 3 Department of Pathology and Laboratory Medicine, IWK Health Centre, Halifax, CAN; 4 Department of Hematology and Oncology, IWK Health Centre, Halifax, CAN

**Keywords:** acute myeloid leukemia (aml), intussusception, myeloid sarcoma, pediatric, small bowel malignancy

## Abstract

A 10-month-old male infant presented to the emergency department with acute abdominal pain. Diagnostic images showed bowel intussusception. A laparotomy with a reduction of ileocolic intussusception and a small bowel resection was done. Initial complete blood count (CBC) showed significant abnormalities of blasts and pancytopenia. Flow cytometry, surgical pathology, and molecular testing confirmed the diagnosis of myeloid sarcoma (MS)/acute myeloid leukemia (AML). The patient was treated as per the Children's Oncology Group (COG) study AAML 1831, low-risk stratification Arm B (LR2), and was treated with five cycles of chemotherapy. Currently, the patient is in remission one year post-therapy. A literature review of pediatric MS patients with presentation of intussusception is included.

## Introduction

Myeloid sarcoma (MS) is a rare manifestation of acute myeloid leukemia (AML), defined as an extramedullary tumor composed of immature myeloid precursor cells [1]. MS may arise concurrently with AML or present as an isolated lesion. Multiple anatomical sites have been reported, including the orbit, central nervous system, lung, bone, abdominal cavity, soft tissue, and skin [2]. In children, intussusception most often occurs without an identifiable cause and is typically managed non-surgically. The mass effect from a tumor may act as a pathological lead point for intussusception. In a single-center study, lymphoma was reported as the most frequent etiology in this setting [3]. Pediatric cases of MS presenting as bowel intussusception are exceedingly uncommon and may be overlooked. Because of its potential for severe clinical consequences, prompt recognition of MS/AML is critical, and systemic chemotherapy remains the cornerstone of treatment. Here, we describe a case of MS associated with AML presenting as bowel intussusception, along with a review of the literature.

## Case presentation

A 10-month-old boy who was previously healthy presented to the emergency department (ED) with a seven-day history of vomiting, abdominal pain, and lethargy. X-ray demonstrated findings suggestive of a small bowel obstruction. The ultrasound confirmed an ileocolic intussusception, with a cluster of enlarged lymph nodes.

The initial complete blood count (CBC) in ED illustrated a significant abnormality of pancytopenia with blasts (Table 1). No Auer rods or acute promyelocytic leukemia (APL) histological features, such as bilobed nuclei, were present.

**Table 1:** Initial CBC The initial CBC in ED illustrated a significant abnormality of pancytopenia with blasts. CBC, complete blood count; RBC, red blood cell; WBC, white blood cell *Manual platelet estimate

Parameters	Patient Values	Reference Range
Blasts	54%	0-0%
WBC	58.42 × 10^9^/L	5.98-13.51 × 10^9^/L
Hemoglobin	71 g/L	101-125 g/L
RBC	2.64 × 10^12 ^/L	4.03-5.07 × 10^12^/L
Platelets	20-50 × 10^9 ^/L*	206-445 × 10^9^/L

Given the unusual CBC findings, it was felt that an underlying hematologic malignancy was likely. Due to this, the surgical team, in consultation with the hematology team, felt that radiologic attempts at reduction of the intussusception would be contraindicated due to increased risk of complications, as well as high suspicion of a pathologic lead point. 

**Figure 1:**
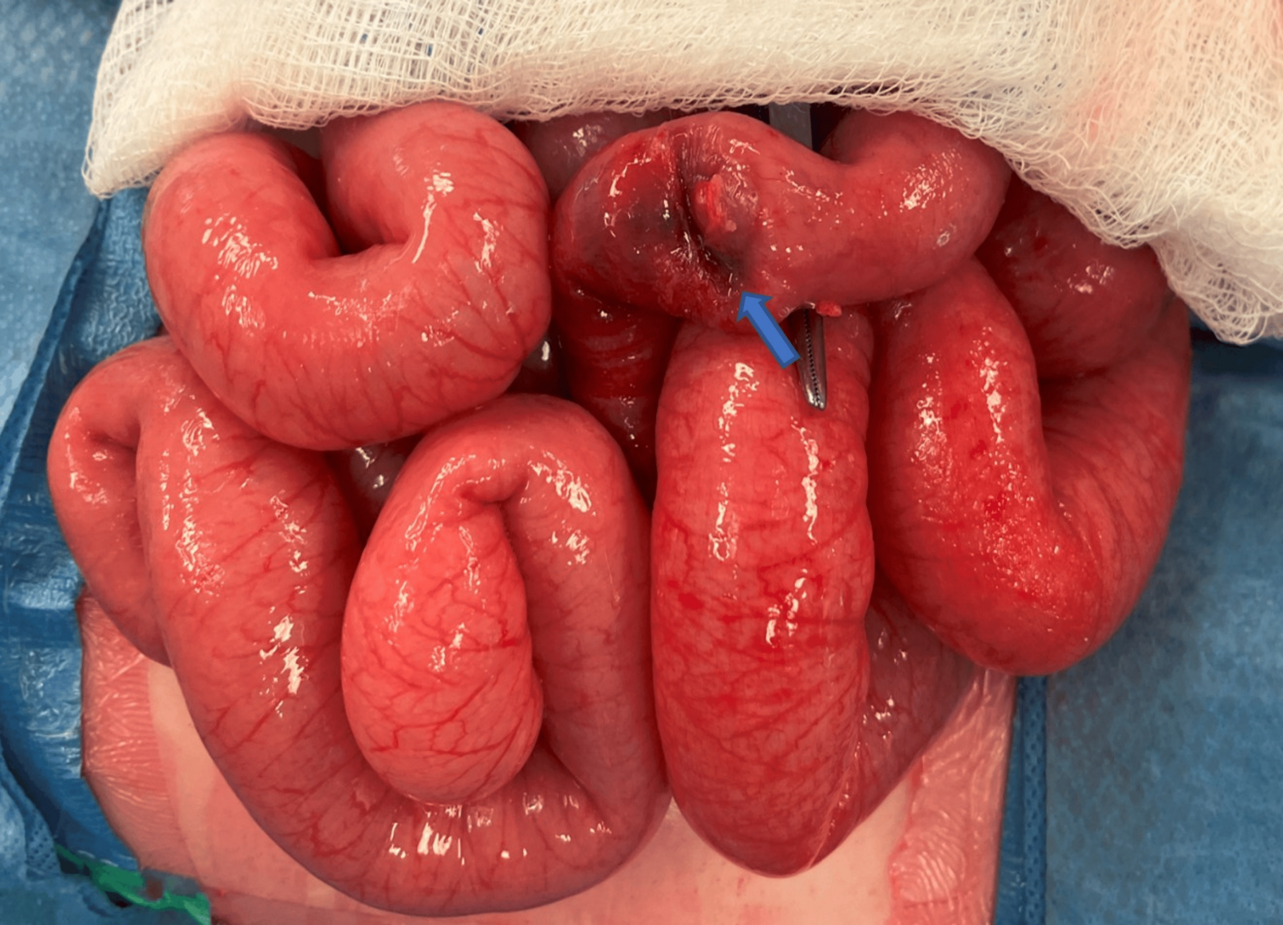
Intro-operative image of the intussusception A dusky, edematous, and congested segment of bowel is noted (arrow), located approximately 10 cm from the ileocolic valve.

An emergency laparotomy was performed with a reduction of ileocolic intussusception and identification of a pathologic lead point within the small bowel (Figure 1). The intussusception portion of the bowel was congested and edematous. There was no perforation identified. The remaining small bowel demonstrated excellent blood supply and appeared healthy, so a primary stapled anastomosis was performed.

**Figure 2:**
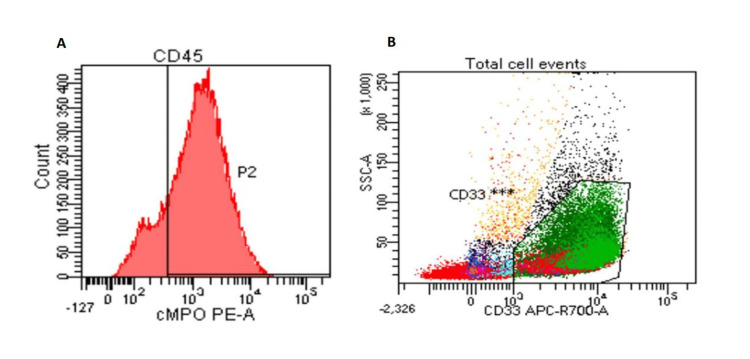
Flow cytometry of the peripheral blood Flow cytometry of the peripheral blood shows a population of blasts that are positive for myeloperoxidase (MPO) and negative for CD45 (A), positive for CD33 (B).

Peripheral blood (Figure 2) and bone marrow flow cytometric analysis showed the blasts (about 65%) that were MPO+, CD33+, and negative for the B-cell and T-cell markers. Bone marrow aspiration and biopsy showed markedly hypercellular with packed blasts. There were rare megakaryocytes and erythroid elements. A diagnosis of AML was raised.

**Figure 3:**
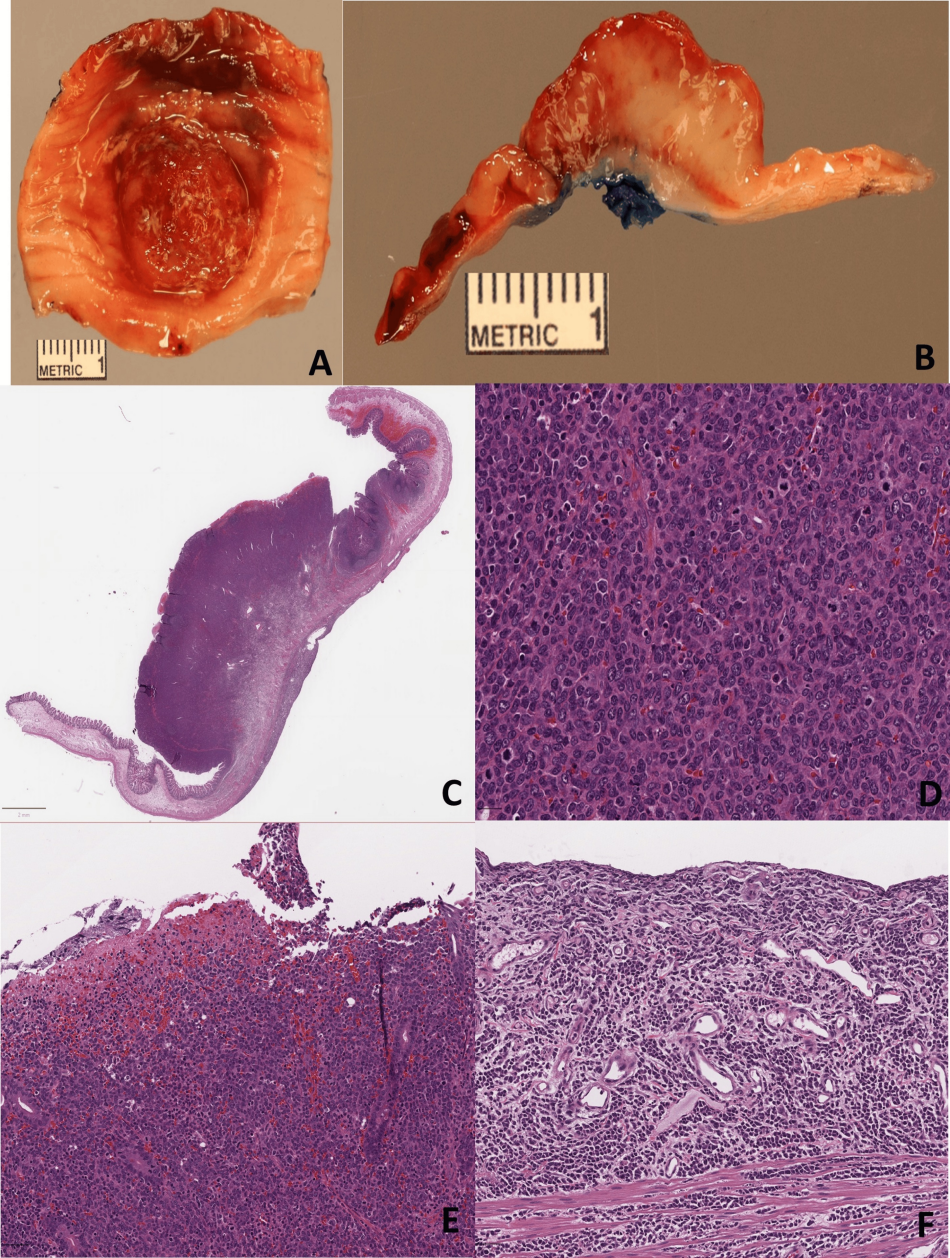
Macroscopic and microscopic images Gross examination revealed a fungating, tan-red, firm mass on the mucosal surface (A), which is solid on the cut surface (B&C). Microscopically, a hypercellular lesion is underneath the mucosa, composed of a population of moderately sized, irregularly shaped cells with a high N: C ratio. They have coarse chromatin, prominent nucleoli and some visible nuclear grooves (D). The surface mucosa is eroded (E). Tumor cells are infiltrating through the muscularis propria and serosa (F).

Histopathology of the small bowel resection showed the bowel mucosa was replaced by a population of moderately sized, irregularly shaped cells with coarse chromatin, prominent nucleoli, and some with visible nuclear grooves, which were consistent with the diagnosis of AML (Figure 3).

Genetic testing showed a KMT2A rearrangement. Molecular tests were negative for FLT3-ITD or FLT3-TKD D835 variant and negative for RUNX1-RUNX1T1 fusion transcript associated with t (8; 21).

The patient was treated as per the Children's Oncology Group (COG) study AAML 1831. Based on negative measurable residual disease (MRD) at the end of induction, cytogenetics, he was on the low-risk stratification Arm B (LR2) arm of the study. He was treated with five cycles of chemotherapy. Currently, the patient is in remission one year post-therapy.

## Discussion

MS is a rare hematological malignancy. It presents as an extramedullary tumor mass of neoplastic myeloid blasts that are considered a tissue-based manifestation of AML, transformed myelodysplastic syndrome (MDS), myeloproliferative neoplasm (MPN), or MDS/MPN [4]. Other myeloid neoplasms, such as chronic myeloid leukemia (CML), chronic myelomonocytic leukemia (CMML), or polycythemia vera (PV), can also be the underlying disease [1].

Patients with MS presenting with intussusception are rare. We did a literature review of pediatric MS patients with the presentation of intussusception (in PubMed/Medline and Embase). Two English literatures were identified (Table 2). Gupta et al. [5] reported a 16-year-old male with no significant past medical history who presented with colocolic intussusception. Histopathology and immunohistochemistry (IHC) revealed MS in the hemicolectomy specimen. Bone marrow and peripheral blood revealed the diagnosis of chloroma. This patient received three cycles of cytarabine and daunorubicin chemotherapy and was doing well one year after surgery. The other case was reported by Kardos et al. [6]. A 2.5-year-old male with neurodevelopmental delay and recent COVID-19 infection presented with appendiceal intussusception. Histopathology and IHC revealed MS in the appendix. The peripheral blood flow cytometry revealed acute monocytic leukemia (AML-M5) with 13% atypical promonocytes/monoblasts. He received ongoing chemotherapy “according to the oncological protocol” when the case was reported. Including the index patient, three of the reported patients are all male. Two patients were previously healthy; one patient had a neurodevelopmental delay, which might increase susceptibility to certain cancers. The neurodevelopmental delay was not specified by the author. This patient and the index patient were reported to have peripheral AML. The molecular studies of the index patient showed KMT2A rearrangement but no evidence of the RUNX I-RUNX III fusion transcript associated with t(8;21) and negative for a FLT3-ITD or a FLT-TKD D835 variant. There are no cytogenetic or molecular analyses available for the two reported intussusception-MS patients.

**Table 2:** Summary of known pediatric myeloid sarcoma (MS)/acute myeloid leukemia (AML) cases with intussusception

Cases	Age/Sex	Past medical history	Site of intussusception	Cytogenetics	Treatment	Outcome
Gupta et al. [5]	16 years/male	None	Colocolic	N/A	Surgery and chemotherapy	Well, one year post-treatment
Kardos et al. [6]	2.5 years/male	Neurodevelopmental delay and recent COVID-19 infection	Appendiceal	N/A	Surgery and chemotherapy	Ongoing chemotherapy when published
Index patient	10 months/male	None	Ileocolic	KMT2A rearrangement	Surgery and chemotherapy	Well, one year post-treatment

The pediatric and adult MS patients have shown differences and overlaps in molecular and cytogenetic features. In adults, mutations in NPM1, FLT3-ITD, PTPN11, KIT, TET2, and NRAS are associated with AML extramedullary disease [7,8]. Cytogenetic abnormalities such as t(8;21) (q22;q22)/RUNX1:: RUNX1T1, CBFβ::MYH11, ETV6::MECOM, FUS::ERG, and PICALM::MLLT10 are also identified [9,10]. In the pediatric patient population, studies found t(8; 21) was associated with MS [11], and higher levels of RUNX1/RUNX1T1 transcripts are risk factors for poor relapse-free survival (RFS) in MS patients [12]. However, Xu et al’s study showed there were no differences between the MS and non-MS groups regarding t(8:21) and FLT3 internal tandem duplication (ITD), NPM1, and CEBPA mutations. In addition, they found that KMT2A rearrangement had a high rate in the MS group [13].

The studies have shown contradicting conclusions regarding the prognostic significance of pediatric MS. Some studies conclude that MS is associated with an unfavorable prognosis in children with AML, while others show MS is related to favorable outcomes or no prognostic significance. Xu et al. studied 884 pediatric patients with AML, including 109 patients (12.3%) who were diagnosed with MS. This study found that the presence of MS and positive KMT2A rearrangement was associated with poor prognosis in terms of event-free survival (EFS) and overall survival (OS) [13]. Johnston et al. [14] analyzed 1459 pediatric AML patients, including 19 patients with CNS-MS and 23 orbital-MS. They found patients with MS had a significantly better survival (complete remission (CR)) rate than those with non-MS and non-CNS-MS [14]. Kobayashi et al. studied 240 pediatric de novo AML, including 56 (23.3%) “extramedullary infiltration (EMI) and found the OS and EFS showed no difference between patients with or without EMI [15]. The discrepant findings in the literature may be due to many reasons. The total case number is relatively small secondary to the rarity of MS. In addition, most cohorts included patients with mixed clinical pictures, such as the sites of MS, isolated MS, or MS with AML, secondary AML/MS post-chemotherapy, or genetic profiles of tumor and bone marrow. 

KMT2A-rearranged AML involves chromosome rearrangement of the KMT2A gene 11q23 [16]. KMT2A fusions are most common in infants with AML [17]. KMT2A-rearrangements involve multiple partner genes and are associated with heterogeneous prognoses. Risk-group stratification has been the focus of numerous studies. The International Berlin-Frankfurt-Münster Study Group (I-BFM-SG) identified independent associations of 4q21/KMT2A::AFF1, 6q27/KMT2A::AFDN, 10p12/KMT2A::MLLT10, 10p11.2/KMT2A::ABI1, and 19p13.3/KMT2A::MLLT1 with adverse outcomes. Importantly, 1q21/KMT2A::MLLT11 was reclassified from favorable to intermediate risk. Additional cytogenetic aberrations (ACAs), including monosomy 10, trisomies 1, 6, 16, and X, add(12p), and del(9q), were also associated with independent adverse prognoses [18]. The genetic study of the index patient demonstrated a KMT2A rearrangement, although the identity of the fusion partner was not determined. Most of the available studies are limited by small cohort sizes. Future risk-stratification efforts for KMT2A-rearranged AML will benefit from integration of advanced methodologies such as MRD detection by flow cytometry and next-generation sequencing (NGS).

Most infant intussusceptions are idiopathic. The reported risk factors include infections, Meckel’s diverticulum, and altered intestinal motility, among others. Intussusception occurs more frequently in boys than in girls, with a ratio of approximately 3:1 in the general population [19]. The typical triad of intermittent abdominal pain, currant jelly stool, and sausage-shaped mass is not very common. However, intermittent abdominal pain, nonbilious emesis, and bloody stools are common. Altered mental status or lethargy can be seen in younger and severe patients [20]. In patients with AML or MS, additional hematologic manifestations may be observed, such as fever, fatigue, mucocutaneous bleeding, and cytopenia, often accompanied by circulating blasts on CBC. Intussusception typically presents with acute gastrointestinal symptoms. In contrast, hematologic-related constitutional manifestations may be subtle or nonspecific, particularly in young children. Therefore, AML or MS should be considered in the differential diagnosis of patients presenting with intussusception.

## Conclusions

While intussusception is the most common pediatric abdominal emergency and is typically prioritized in the differential diagnosis, the presence of systemic or atypical symptoms should prompt further evaluation. Pediatric AML or MS presenting as an intussusception is very rare, which poses challenges in diagnosis and can lead to delayed chemotherapy treatment, as surgery is needed. AML/MS should be included as a differential diagnosis in patients with a suspicious tumor, or in patients presenting with intussusception, in addition to markedly abnormal hemoglobin, platelet, and white blood cell counts. In these cases, thoughtful discussion between surgery and hematology is warranted. Surgical management should be considered rather than standard radiologic reduction of the intussusception.
